# COVID-19 Pneumonia and Increased Insulin Requirement in Known Diabetic Patients: A Prospective Observational Study

**DOI:** 10.7759/cureus.50239

**Published:** 2023-12-09

**Authors:** Elangkumaran V Manoharan, Nandeeswaran Kola Sridharan, Balaji Kesavan, Geront A Andrews, Gowtham Sundaram Venkatesan, Priyanga Kesavan

**Affiliations:** 1 General Medicine, Kovai Medical Center and Hospital, Coimbatore, IND; 2 Anaesthesiology and Critical Care, Meenakshi Mission Hospital and Research centre, Madurai, IND; 3 Anaesthesiology, University Hospital of North Tees, Stockton-on-Tees, GBR; 4 Anaesthesiology and Critical Care, Rela Institute and Medical Centre, Chennai, IND; 5 General Surgery, James Cook University Hospital, Middlesbrough, GBR; 6 Dentistry, Kovai Medical Center and Hospital, Coimbatore, IND

**Keywords:** management of diabetes in covid, hyperglycaemia in covid, insulin requirements in covid, insulin resistance in covid, covid-associated diabetes, diabetes management in covid 19, covid-19 and diabetes

## Abstract

Background

COVID-19-related critical illness affects multiple organs and causes a variety of metabolic derangements in the body's physiology that are not proven with the current level of evidence. Insulin resistance and deranged blood sugar control due to COVID-19 have been major problems when managing diabetic patients with hyperglycaemia when they are admitted with COVID-19 pneumonia. There is a lack of abundant literature to prove the excess insulin requirements of COVID-19 and to quantify their insulin needs scientifically. This study aims to quantify the degree of insulin dose increments in these patients.

Materials and methods

The study is a single-centre prospective observational study done in COVID-19 wards at a tertiary care hospital in India. The diabetic patients admitted with COVID-19 pneumonia between June 2020 and December 2020 were included in the study. Seventy-five patients with fair control of diabetes (HbA1C <7.5) were included in the study. Their average daily insulin requirement was calculated for the first seven days of admission. This was tabulated and compared to their baseline insulin requirement before being unwell due to COVID-19. A sub-group analysis was also done to show the relation between severity of illness and glycaemic dysregulation.

Result

Invariably, all patients were found to be hyperglycaemic on admission. Insulin need has increased to 1.5 to 2.5 times the baseline values in the first 24 hours of admission. This insulin dose requirement stayed high around the same levels for all seven days of observation. The average mean value of the daily insulin dose for the seven days of study was calculated to be 132 units. This is more than twice the mean baseline daily insulin requirement of 62 units during the pre-COVID-19 period. Subgroup analysis showed that the severe group had poor glycaemic control, requiring higher doses compared to their own baseline and also to the moderate group.

Conclusion

COVID-19 pneumonia significantly increases insulin resistance and insulin requirements during illness in fairly controlled known diabetic patients with insulin. Managing this COVID-19-induced hyperglycaemia requires 1.5 to 2.5 times the baseline insulin doses.

## Introduction

The COVID-19-related critical illness affects multiple organs and causes a variety of metabolic derangements in the body's physiology, which are not proven with the current level of evidence [1,2]. Insulin resistance and deranged blood sugar control due to COVID-19 have been major problems when managing diabetic patients with hyperglycaemia when they are admitted with COVID-19 pneumonia [1]. The complex mechanisms underlying these increased insulin requirements for blood sugar control have been hypothesized in various published literature but have not been proved by molecular analysis to date [2]. As COVID-19 infection has been known worldwide only in the recent few years, the holistic management of patients with COVID-19 infection does not have established guidelines or recommendations.

In our centre, we found it increasingly difficult to manage blood sugar within the normal limits (70 to 140 mg/dl) in patients with COVID-19 pneumonia. Practical recommendations for managing diabetes in COVID-19 were published in the Lancet in June 2020 [3]. The requirements for insulin were high, up to more than three to six times the normal daily doses of insulin they would otherwise require on a normal day.

Research on COVID-19 shows growing interest in how COVID-19 impacts the regulation of blood glucose levels, and some studies propose that COVID-19 could be a virus that uniquely promotes the development of diabetes [4]. Nevertheless, there is a lack of abundant literature to prove the excess insulin requirements during COVID-19 and to quantify their insulin needs scientifically. This study aims to fill this research gap by quantifying the insulin resistance caused by COVID-19 pneumonia and measuring the degree of insulin dose increments in these patients. These quantitative observations can be the basis for future research on the pathophysiology of COVID-19 pneumonia and its relation to insulin resistance.

## Materials and methods

Aims and objectives

The observational study aimed at measuring the poor glycaemic control quantitatively in patients with COVID-19 infection admitted to the hospital. This was done by comparing the magnitude of the increase in daily total insulin requirements compared to their baseline insulin requirements in known diabetics with fair control.

Methodology

The study is a single-centre prospective observational study done in COVID-19 wards at Kovai Medical Center and Hospital, a tertiary care hospital in India. The diabetic patients admitted with COVID-19 pneumonia to the hospital between June 2020 and December 2020 were included in the study. Admission criteria for patients infected with the COVID-19 virus is the presence of respiratory distress or hypoxia requiring treatment. Asymptomatic patients are not admitted to conserve resources during the pandemic. Detailed inclusion and exclusion criteria are described in Table 1. No intervention was done for the purpose of the study, nor is there any control arm to compare, which is not feasible in the given context. A pilot observation was done in the months of April and May 2020, which included 15 patients, to look for consistent increased insulin requirements in patients with COVID-19 pneumonia. The data from these 15 patients is not included in the main study. The data collection was started in June 2020 with the help of a questionnaire. No patient identification details were recorded in the questionnaire sheet. The Ethics Committee of Kovai Medical Center and Hospital approved the study (ECR/112/Inst/TN/2013).

**Table 1:** Patient inclusion and exclusion criteria for the study HbA1C: glycosylated haemoglobin which shows the average glucose control over the past three months, DKA: diabetic ketoacidosis

Inclusion criteria	Exclusion criteria
Age >18 years	Patient refusal
Patients with COVID-19 pneumonia needed admission to hospital	Newly diagnosed diabetes during that hospital admission
Known diabetic (includes all types)	Poorly controlled diabetes HbA1 C >7.5
Admission HbA1C <7.5	Patients requiring glucose-insulin infusions, as intolerable to oral feeds/DKA
	Intubated patients and patients fed on nasogastric feeds
	Patients with severe end-organ damage related to diabetes like nephropathy, retinopathy

A total of 123 patients met the inclusion criteria. Informed consent was obtained from these patients. After recruitment, patients were followed up for seven days after admission. Within these seven days, after recruitment, some patients worsened, requiring invasive ventilation, and were shifted to the critical care unit. A few patients were discharged from the hospital or transferred to other hospitals. They were excluded from the study. The data was extracted from the diabetic logbook sheet, which is a hospital record attached to the patient file. This gives the values of date, time, blood sugar, and insulin prescribed/administered, as well as the changes made in the comments section.

If the levels of HbA1C are greater than 7.5, it is evident that their routine doses of insulin were inadequate even before the COVID-19 infection. We do not know the baseline insulin requirement for better control of diabetes in these patients. Thus, they were excluded from the study.

As per the local hospital policy, all patients on oral hypoglycaemic agents were converted to equivalent doses of insulin targeted at normal blood glucose levels. Poor oral intake due to respiratory distress, the risk of hypoglycemia and lactic acidosis associated with oral hypoglycaemic agents, and the need to avoid interruption of non-invasive ventilation (NIV) were the reasons guiding the hospital's policy to switch all patients from oral agents to insulin. Additionally, insulin titration is easier and more predictable and allows tailoring based on blood glucose levels. The hospital provides a separate diet appropriate for diabetic patients; therefore, the bias due to the dietary influence on acute hyperglycaemia can be safely ruled out.

HbA1C levels are checked on admission to the hospital. Their daily insulin dose requirements are noted, which is the baseline for comparison with the insulin needs after COVID-19 infection. The insulin requirement includes all types of insulin, irrespective of whether they are long, intermediate, or short-acting. Only the total units of insulin required cumulatively over the day are recorded. On admission, to start with, these patients had been prescribed the routine doses of insulin they needed when they were normally well before the COVID-19 infection. The patients' capillary blood glucose was monitored six times a day: fasting, two hours post-breakfast, pre-lunch, two hours post-lunch, pre-dinner, and two hours post-dinner. The targeted blood glucose was 70 to 120 for pre-meal values and 100 to 160 for two-hour post-meal values. During the COVID-19 pandemic, there was decreased manpower and compromise in the nurse-patient and doctor-patient ratios. Frequent hourly contact with the patients is avoided for the risk of spreading infection. Poor oral intake due to respiratory distress and the need for an NIV all pose the risk of hypoglycaemia in these critically ill patients. For all these reasons, a higher limit of glucose targets of 120 for pre-meal and 160 for post-meal is intentionally set in these patients. The basal insulin was prescribed once a day for long-acting insulin or twice a day for intermediate-acting insulin. No change in the patient’s insulin type or preparation was made. The postprandial hyperglycaemia was treated with rapid and short-acting insulin. When the total dose of this rapid/short-acting insulin per day exceeds 20 units, it is converted to equivalent intermediate/long-acting insulin, appropriately divided and added to the morning and evening doses.

The data extracted is entered into an Excel worksheet (Microsoft Corporation, Washington, USA). Daily insulin doses are added together every day for seven days. The mean of seven values is calculated. The total mean of all the patient's insulin requirements for that specified day is calculated. The multiple changes from the baseline daily insulin dose are measured and recorded. Charts are made from Microsoft Excel for a visual representation of the magnitude of the rise in total daily insulin requirements.

## Results

Out of 123 patients, 44 patients were excluded or dropped out for various reasons like early discharge, worsening to invasive ventilation, death, or incomplete data from the diabetic logbook sheet. Four patients had diabetic ketoacidosis, requiring insulin therapy based on the standard diabetic ketoacidosis protocol. Data from the rest of the 75 patients were recorded and analyzed. Out of 75 patients, 44 were males and 31 were females. The mean age of the patients was 56 +/- 8.2 years. Sixty-seven out of 75 patients were type 2 diabetics on oral hypoglycaemic agents and/or insulin; the rest were type 1 diabetics. The baseline insulin requirement was recorded. The mean daily insulin requirement of the patients was 62 units +/- 6.7. The total insulin dose required after admission is recorded day by day. The percentage of increase from the baseline has been calculated and recorded as multiples from the baseline. The average daily insulin requirement for the first seven days has been calculated and tabulated in Table 2.

**Table 2:** Mean and range of daily insulin requirement

	Mean value of daily insulin dose (in units) requirement	Multiples of baseline value on specified day	Range of insulin requirement (in units) on specified day	
			Lowest	Highest
Pre-COVID-19	62	1.0	40	86
Day 1	118	1.9	86	156
Day 2	126	2.0	84	165
Day 3	136	2.2	90	170
Day 4	138	2.2	100	182
Day 5	129	2.1	89	168
Day 6	148	2.4	101	202
Day 7	130	2.1	95	185
Total	132	2.1	90	175

Invariably, all patients were found to be hyperglycaemic on admission. None of the patients in the study had blood glucose control with their baseline insulin doses. The insulin dosage needed within the initial 24 hours was 50% to 150% higher than the baseline (range 86 to 156 units). Calculating from the above-mentioned baseline insulin value of 62 units, this has increased to 1.5 to 2.5 times the baseline values in the first 24 hours of admission. The average mean value of the daily insulin dose for the seven days of study was calculated to be 132 units, as shown in Table 2. This is more than twice the mean baseline value (62 +/- 6.7 units) for daily insulin requirement compared to the pre-COVID-19 period. This insulin dose requirement stayed high at around the same levels for all seven days of observation, as shown in Table 2. The highest recorded daily insulin requirement during these 7 days was 202 units for a patient on his sixth day of hospital admission.

A subgroup analysis was made based on the severity of COVID-19 pneumonia. Patients who are severely hypoxic with oxygen saturations below 88% on room air and those who are tachypnoeic and in distress requiring NIV support are grouped in the severe category. Patients who have room air saturations above 88% and who have happy hypoxia are grouped in the moderate category. This sub-grouping of the patients was easy, as they were admitted to different wards in the hospitals for medical administrative logistics. Few patients have moved across the category during the study. However, the outcomes remain unaffected, given that only the mean daily insulin requirement was studied.

Table 3 shows subgroup analysis results and helped postulate that the amount of insulin needed to control hyperglycaemia paralleled the severity of COVID-19 illness. In the first seven days of admission, severe COVID-19 patients require more than two times the insulin doses they would normally require. The severe group had poor glycaemic control, requiring higher doses compared to their own baseline and also to the moderate group, as seen by the values in Table 3.

**Table 3:** Subgroup analysis showing the insulin requirement parallels the severity of COVID illness

	Multiples of daily insulin doses required with ranges in the bracket	
	Moderate illness	Severe illness
Day 1	1.5 (72-110)	2.0 (88-156)
Day 2	1.5 (84-103)	2.1 (100-165)
Day 3	1.6 (90-112)	2.2 (108-170)
Day 4	1. 8 (100-127)	2.4 (113-182)
Day 5	1.8 (89-130)	2.4 (124-168)
Day 6	2.0 (101-148)	2.7 (128-202)
Day 7	1.8 (95-133)	2.3 (103-185)

## Discussion

The severe hyperglycaemia on admission itself is a marker of dysregulated glucose-insulin metabolism in patients with COVID-19 infections [3]. Our study results show that COVID-19 infection severe enough to require hospital admission causes an increased need for insulin than the routine daily dose that would be needed otherwise in patients with fair diabetic control. This ranges between 1.5 and 2.7 times the baseline dosage based on the severity of COVID-19. The target for blood glucose control in COVID-19 pneumonia has not been well defined to date. Numerous reviews have already outlined practical methods for managing elevated blood sugar levels in COVID-19 cases [3-5], one of which is the practical guidelines from the Lancet Diabetes and Endocrinology journal [4]. At present, there is no compelling reason to implement new strategies or establish blood sugar level targets for treating hyperglycaemia in COVID-19 when compared to other viral pneumonia. Therefore, in our study, we adopted the local hospital policy, considering other practical difficulties during the COVID-19 pandemic.

The mechanism of uncontrolled hyperglycaemia during COVID-19 infection is unclear. Whether the COVID-19 virus affects the pancreas to cause absolute insulin deficiency or works by mechanisms to increase insulin resistance at the cellular level is not proven by the currently available literature [6,7]. Studies supporting either of these mechanisms have been published. Studies supporting insulin deficiency have demonstrated various findings, like elevated pancreatic enzymes, direct cytolytic beta cell damage causing loss of function, or pancreatitis features on post-mortem analysis [6-8]. Studies supporting insulin resistance for COVID-19-induced hyperglycaemia have demonstrated hyperinsulinemia and elevated C-peptide levels [9].

A key molecular connection between the severity of COVID-19 and insulin resistance is likely the angiotensin-converting enzyme (ACE), which is abundantly found in pancreatic beta cells [10]. This enzyme serves as the receptor through which coronaviruses like SARS-CoV-2 attach to their target cells [11]. Blocking or inhibiting the ACE2 receptor leads to a significant increase in angiotensin 2 levels and the hyperactivity of the renin-angiotensin-aldosterone system, resulting in elevated oxidative stress and reduced sensitivity to insulin [12]. It has been suggested that SARS-CoV-2, the virus responsible for COVID-19, might directly impact the functioning and survival of pancreatic β-cells, which can lead to a rapid and severe deterioration of metabolic control in individuals with pre-existing diabetes or result in the onset of new diabetes cases [13]. In support of these concepts, our study results show very high insulin doses, which proves the severe impact of the COVID-19 virus on glycaemic regulation, as shown in Table 2.

Prior research indicates that the elevated blood sugar levels observed in COVID-19 patients are temporary and tend to normalize during the recovery period [14]. However, a six-month prospective study by Chen et al. suggests that COVID-19 may increase the risk of insulin resistance even in patients without pre-existing diabetes, even after recovery [15]. However, there has been a note from the results of this study about decreased insulin secretion as a cause of hyperglycaemia in patients with COVID-19 infection. As per a retrospective cohort study by Lockhart et al., the excess insulin requirement in severe COVID-19 compared to non-COVID-19 viral pneumonitis is related to the severity of respiratory failure and pre-existing diabetes [14]. Our subgroup analysis also proves that the degree of insulin resistance is directly proportional to the severity of the illness. Earlier research indicates that individuals critically ill with both diabetes mellitus and COVID-19 exhibited elevated insulin needs and experienced a less favourable duration within the target blood glucose range during the peak of the inflammatory response [16]. Our observations align with these findings: the demand for insulin corresponds to the severity of COVID-19.

Reports from Rubino et al. to the New England Journal of Medicine state exceptionally high insulin doses are required to treat diabetes-related hyperglycaemia and its complications in patients with pre-existing diabetes. In response to these concerns, a consortium of prominent diabetes researchers from around the world involved in the CoviDIAB Project has initiated a worldwide registry specifically for individuals with hyperglycaemia related to COVID-19. This registry can be accessed at covidiab.e-dendrite.com. Though this registry initially included only new-onset diabetes following COVID-19 infection, it has now been expanded to include patients with pre-existing diabetes who exhibit severe insulin resistance. Analysis of worldwide data from this registry is expected to provide more information regarding the cause or association between COVID-19 infection and dysregulated glycaemic control [17,18].

Our study is a small contribution to the COVID-19 literature to prove the association between deranged glycaemic control during acute illness. More studies are required targeting the mechanisms causing this hyperglycaemia, which will help in managing diabetes during COVID-19 pneumonia.

Limitations

Our study had potential limitations. Insulin requirements after recovery from COVID-19 were not studied if there was a return to baseline. This is due to the restrictions on hospital visits during the pandemic. At discharge, the patients are advised to follow up on their blood glucose using a glucometer and adjust the insulin dose accordingly, as per the diabetic handbook given to them. There was no long-term follow-up done to check the HbA1c levels. This was particularly difficult due to the COVID-19 lockdown and restricted hospital visits. This is a single-centre study, and the association between steroids used for COVID-19 and hyperglycaemia was not studied.

## Conclusions

COVID-19 pneumonia significantly increases insulin resistance and insulin requirements during illness in fairly controlled known diabetic patients with insulin. Managing this COVID-19-induced hyperglycaemia requires 1.5 to 2.5 times the baseline insulin doses. More evidence is required to prove the mechanisms behind COVID-19-induced hyperglycaemia, its management guidelines, and long-term follow-up.
